# Genome-wide transcriptional response of *Trichoderma reesei* to lignocellulose using RNA sequencing and comparison with *Aspergillus niger*

**DOI:** 10.1186/1471-2164-14-541

**Published:** 2013-08-09

**Authors:** Laure Ries, Steven T Pullan, Stéphane Delmas, Sunir Malla, Martin J Blythe, David B Archer

**Affiliations:** 1 School of Biology, University of Nottingham, Nottingham NG7 2RD, UK; 2 Université Pierre et Marie Curie (UPMC, Université Paris 06), Sorbonne Universités, UMR 7138, Systématique Adapation et Évolution, 75005 Paris, France; 3 Deep Seq, Centre for Genetics and Genomics, Queen’s Medical Centre, University of Nottingham, Nottingham NG7 2UH, UK

**Keywords:** *Trichoderma reesei*, *Aspergillus niger*, Glycoside hydrolases, Carbohydrate esterases, Antisense RNA, RNA-sequencing

## Abstract

**Background:**

A major part of second generation biofuel production is the enzymatic saccharification of lignocellulosic biomass into fermentable sugars. Many fungi produce enzymes that can saccarify lignocellulose and cocktails from several fungi, including well-studied species such as *Trichoderma reesei* and *Aspergillus niger*, are available commercially for this process. Such commercially-available enzyme cocktails are not necessarily representative of the array of enzymes used by the fungi themselves when faced with a complex lignocellulosic material. The global induction of genes in response to exposure of *T. reesei* to wheat straw was explored using RNA-seq and compared to published RNA-seq data and model of how *A. niger* senses and responds to wheat straw.

**Results:**

In *T. reesei*, levels of transcript that encode known and predicted cell-wall degrading enzymes were very high after 24 h exposure to straw (approximately 13% of the total mRNA) but were less than recorded in *A. niger* (approximately 19% of the total mRNA). Closer analysis revealed that enzymes from the same glycoside hydrolase families but different carbohydrate esterase and polysaccharide lyase families were up-regulated in both organisms. Accessory proteins which have been hypothesised to possibly have a role in enhancing carbohydrate deconstruction in *A. niger* were also uncovered in *T. reesei* and categories of enzymes induced were in general similar to those in *A. niger*. Similarly to *A. niger*, antisense transcripts are present in *T. reesei* and their expression is regulated by the growth condition.

**Conclusions:**

*T. reesei* uses a similar array of enzymes, for the deconstruction of a solid lignocellulosic substrate, to *A. niger*. This suggests a conserved strategy towards lignocellulose degradation in both saprobic fungi. This study provides a basis for further analysis and characterisation of genes shown to be highly induced in the presence of a lignocellulosic substrate. The data will help to elucidate the mechanism of solid substrate recognition and subsequent degradation by *T. reesei* and provide information which could prove useful for efficient production of second generation biofuels.

## Background

The burning of fossil fuels is accompanied by many recognised problems affecting the global economy and environmental health. Replacing fossil fuels with biofuels will help to reduce global CO_2_ emissions, produce a more favourable greenhouse gas profile, decrease dependence on diminishing oil resources and promote local economies [1,2]. The production of biofuels from plant biomass such as grasses [3], woods [4,5] and lignocellulosic wastes [6], does not compete with food production and is called second generation (2G) biofuel [7].

In nature, the degradation of lignocellulosic biomass is catalysed by enzymes from various microorganisms such as saprobic fungi and bacteria [7]. Enzymes for biomass deconstruction, and many other industrial applications, are often derived from fungi from the *Trichoderma* and *Aspergillus* genera [1]. The overall aim of this study was to investigate the strategy that *Trichoderma reesei* employs to degrade complex lignocellulosic polysaccharides and compare this to the mechanism used by *Aspergillus niger* in order to provide novel insights which may prompt the development of new approaches for the production of 2G biofuels.

*Trichoderma* spp. and *Aspergillus* spp. have many industrial applications due to their production of very high levels of secreted enzymes [1,3,8]. This has led to the development of a wide selection of genetic tools in *T. reesei*, including random and targeted mutagenesis to create cellulase hyper-producing mutants [9,10], to elucidate regulatory mechanisms of pathways concerning the metabolism of simple sugars [11,12] and to target the *T. reesei* secretion system in order to produce higher protein yields through engineering more efficient and thermostable enzymes [2,9,13-17]. To date, the genome of *T. reesei* has been found to encode a total of 228 polysaccharide-degrading enzymes that represent 61 enzyme families [18]. This is similar to the total number (ca. 280) of carbohydrate-degrading enzymes in *A. niger*.

This study characterises the transcriptional changes associated with exposure to wheat straw using Next Generation RNA sequencing (RNA-seq) technology, with the aim of gaining an understanding of the steps leading to the deconstruction this complex lignocellulosic substrate. Comparing them to the mechanisms employed by *A. niger* previously described [3], will uncover relevant differences and similarities in lignocellulose degradation between both industrially important organisms. The cost of enzymes presents a major challenge in the cost-effectiveness of biofuel production [19], and the cost of enzymes can be reduced by a combination of factors. Firstly, the yield of enzymes from the fungal source should be maximised and, secondly, the most effective mix of functionalities is required. Other aspects such as the site of production of the enzymes are also important. Ground and autoclaved, but otherwise un-treated, wheat straw was used in this study but a variety of pre-treatments are possible when digesting lignocellulose for the generation of 2G biofuels. That will inevitably alter the fungal responses but an understanding of the mechanistic basis of those responses requires a base-line study with non-pre-treated material, as described here.

## Results and discussion

### The wheat straw –induced transcriptome of *T. reesei* QM6a

The *T. reesei* genome is 33.9 Mb in size with 9,126 predicted genes [20]. Transcriptomes were sequenced from replicated independent cultures under 3 different sets of conditions: 1) after growth from conidia for 48 h in the presence of glucose as sole carbon source, a monosaccharide which represses expression of many genes involved in plant cell wall degradation, 2) 24 h after transfer of washed mycelia from 1) into media containing ground wheat straw as the sole carbon source to monitor the induction of genes involved in polysaccharide deconstruction and 3) 5 h after addition of glucose to the straw cultures from 2) to determine genes responsive to carbon catabolite repression. The ball milled wheat straw used in this study contained 37% cellulose, 32% hemicelluloses, 22 ± 0.1% lignin and was 25% crystalline [3]. Statistical tests [21-23] were applied to enable us to identify all genes which were significantly differentially expressed (p-value of <0.001 for all three tests) between the three conditions studied (see Additional file 1). RPKM values were calculated for each of the biological replicates as well as for the combined mapping of all replicates at 48 h, 24 h straw and 5 h glucose (see Additional file 1). The results shown in this study are from the combined mapping scores and only inductions showing a significant score in all statistical tests are discussed.

### Expression of CAZy genes in *T. reesei* and comparison with *A. niger*

The degradation of plant cell wall carbohydrates is mediated by enzymes of four different classes: the carbohydrate esterases (CEs), the polysaccharide lyases (PLs), the glycoside hydrolases (GHs) and the auxiliary activities (AAs). These enzymes are classed, based on their primary amino acid sequence and related activity, into families in the Carbohydrate Active Enzyme database (CAZy) (http://www.cazy.org) [24]. Analysis of the *T. reesei* QM6a genome identified 22 CE-encoding genes representing 8 families, 5 PL-encoding genes representing 3 families, 195 GH-encoding genes, representing 49 families and 6 AA-encoding genes, representing 1 family [18]. The *A. niger* ATCC 1015 genome contains 25 CEs representing 9 families, 8 PLs representing 2 families, 239 predicted GHs representing 50 families and 7 AAs representing 1 family [25]. There are differences in the families of CEs, PLs and GHs encoded by the genomes of both fungi [18,20,25]. PLs are not as important as GHs and CEs for wheat straw degradation, as PLs mainly target pectin, a structure which is also degraded by enzymes of many GH families, including GH family 28 [26]. The family of AAs encoded by the genomes of *T. reesei* and *A. niger* (AA family 9) were formerly known as GH61s but were shown to be copper-dependent oxidases and have a different catalytic mechanism to the GHs [27]. Enzymes of AA family 9 play important accessory roles in enhancing lignocellulose degradation [28].

Carbohydrate esterases which play a role in lignocellulose degradation and which are encoded by *T. reesei* but not by *A. niger* belong to CE family 15. The genome of *T. reesei* encodes one CE family 15 glucuronoyl esterase [JGI:123940], also known as CIP2, which contains a cellulose binding module (CBM1) and which plays an important role in dissociating lignin from hemicelluloses through targeting the ester bonds between the aromatic alcohols of lignin and the glucuronic acid residues from the xylose backbone in hemicelluloses [29]. CE families 8 and 12 are present in *A. niger* but not in *T. reesei*[18,25] and the genome of *A. niger* encodes 3 CE family 8 pectin methylesterases involved in the de-esterification of pectin [30], and two CE family 12 rhamnogalacturonan acetylesterases involved in the deconstruction of plant cell wall pectin [31].

Glycoside hydrolases involved in lignocellulose deconstruction and encoded by *T. reesei* and not *A. niger* belong to GH families 39, 115 and 45 and assist in the degradation of xylan (GHs 39, 115) and cellulose (GH 45) [18]. In *A. niger*, proteins from GH families 26 and 51, which are not encoded by *T. reesei*, are involved in the degradation of hemicellulosic mannan and arabinan residues [26]. Furthermore, the genome of *A. niger* encodes proteins of GH families 53 and 88, which are involved in the degradation of pectin [25,26,32], inulinases and invertases belonging to GH family 32 and which degrade polysaccharides containing fructose and sucrose [25].

**Figure 1:**
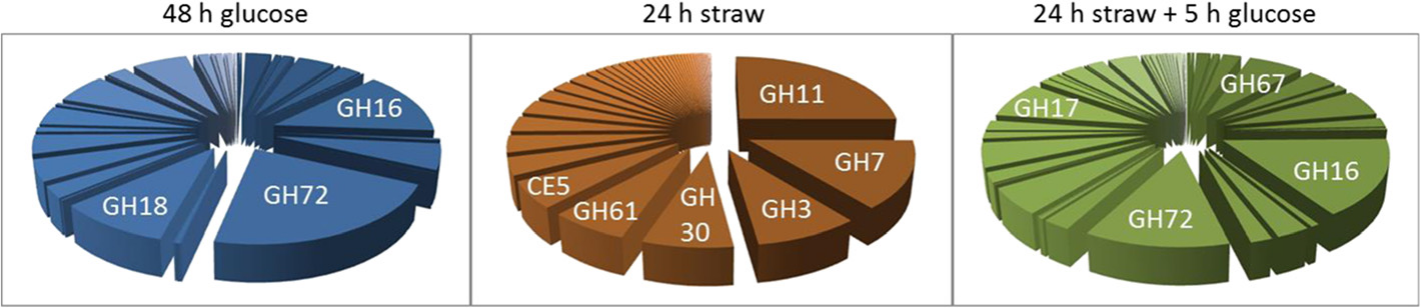
**CAZy gene expression.** Comparison of the percentage of RNA transcripts corresponding to glycoside hydrolases, carbohydrate esterases and polysaccharide lyases in *T. reesei* and *A. niger* when grown for 48 h in glucose, transferred into straw-based media for 24 h and with the addition of glucose to straw-incubated cultures for 5 h. Error bars represent standard deviations of the percentage of RNA transcripts corresponding to GHs, CEs and PLs for all replicates.

**Figure 2:**
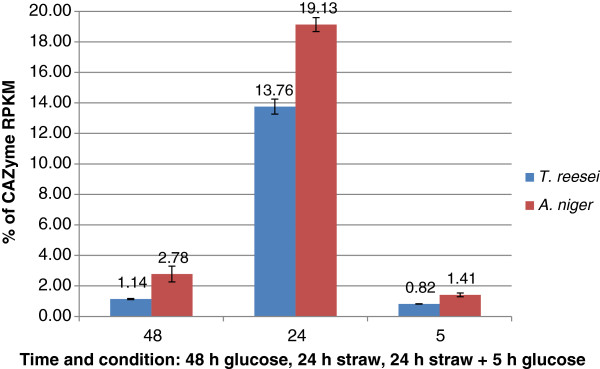
**CAZy enzyme families.** Proportions of total of CAZy gene mRNA from each enzyme family when mycelia were grown in glucose for 48 h, transferred to straw for 24 h and with the addition of glucose to straw-incubated cultures for 5 h. The enzyme families with the highest RNA percentage for each condition are indicated and listed in decreasing order of expression in the 24 h straw conditions.

After 48 h growth in glucose, CAZy gene mRNA represented 1.14% of total RNA in *T. reesei* (c.f. 3% in *A. niger*, Figure 1), with proteins from GH families 16, 18 and 72 (glucanases, chitinases and glucanosyltransferases) representing approximately half (45%) of the total CAZy mRNA (Figure 2). Thus in *T. reesei*, low levels of mRNA from genes encoding enzymes involved in the degradation of complex carbohydrates, including hemicellulose and chitin, are present when the fungus is cultivated in glucose-based medium. In this medium many of these enzymes are likely to be involved in cell wall remodelling during hyphal extension as high growth rates are achieved in the presence of glucose in *T. reesei*[33]. In contrast, in *A. niger*, transcripts from the glucoamylase *glaA* gene accounted for over 65% of total CAZy mRNA in glucose medium [3]. Induction of this gene in the presence of glucose and glucose-containing polysaccharides such as such starch has previously been described [34].

Similarly to *A. niger*, transfer into straw-rich medium for 24 h caused an increase in CAZy gene mRNA in *T. reesei* albeit to a lesser extent than in *A. niger* (13.76% compared to 19% of total cellular mRNA, Figure 1). Thirty-three *T. reesei* CAZy-encoding genes, representing 17 different GH families, 3 CE families, 1 AA family and including two accessory enzymes (SWO1 and CIP1), were transcriptionally up-regulated over 20-fold between 48 h glucose and 24 h straw samples and reached an expression level above 50 RPKM (Table 1). These results are in agreement with previous microarray data, which showed that genes encoding enzymes of GH families 3, 5, 11, 28, 30 and of AA family 9 (Table 1) were induced in *T. reesei* mycelia when grown in the presence of wheat straw [18]. Importance of these enzymes in plant cell wall deconstruction is suggested by the presence of well-characterised hydrolase genes such as those coding for BGL1, BGL2 and CEL1B (Table 1). The genome of *T. reesei* encodes 11 predicted β-glucosidases (GH families 1 and 3) which were shown to be functionally diverse and differently expressed in the presence of various carbon sources [18]. The extracellular *T. reesei* β-glucosidase BGL1 has been described as having transglycosylation activity in the presence of insoluble substrates such as crystalline cellulose, and is therefore possibly involved in cellulase gene induction by generating inducer molecules [11]. Moreover, it was also shown that deletion of the intracellular β-glucosidases BGL2 and CEL1B as well as BGL1 led to a significant delay in *cbh1* (cellobiohydrolase CEL7A) induction highlighting an important role for these enzymes in plant cell wall degradation [35]. Proteins from four GH families (3, 7, 11 and 30) including β-glucosidases, cellobiohydrolase CEL7A and xylanases; from AA family 9 (copper oxidoreductases) and from CE family 5 (acetyl xylan esterases) represented the majority (approximately 65%) of mRNA out of the total CAZy mRNA after 24 h incubation in the presence of straw in *T. reesei* (Figure 2). These proteins are likely to be the main enzymes required for wheat straw degradation. In *T. reesei* and *A. niger*, transcript abundance from genes encoding GH family proteins 3, 5, 6, 7, 11, 30, 31 and 67 (glucosidases, cellobiohydrolases, xylanases and glucuronidases) and AA family 9 (copper oxidoreductases) was highest for both organisms (Table 1) indicating that both species use a similar array of GHs for wheat straw deconstruction. These results were in agreement with results from microarray studies, which showed the induction of genes encoding endoglucanases and mannanases (GH5), cellobiohydrolases (GH6 and 7), β-glucosidases (GH3), xylanases (GH11 and GH30), enzymes of AA family 9 and acetyl xylan esterases (CE5) in *T. reesei* mycelia when cultivated in the presence of wheat straw [18]. The same study described the induction of genes encoding enzymes from GH families 16, 18, 27, 55, 95 and 105 in the presence of wheat straw [18]. This is in contrast to the results presented here and is likely to be the cause of the use of different strains (RUTC30 [18] vs. QM6a), differently pre-treated wheat straw (steam explosion [18] vs. ball milled), different conditions (non-inducing, non-repressing [18] vs. repressing) and the use of different sequencing technologies (microarrays [18] vs. RNA-seq). An advantage of RNA-seq, when compared to microarrays, is that it is not limited to detecting transcripts that correspond to known genomic sequences, background signals are low and it does not have an upper limit for quantification [36]. As a consequence, RNA-seq allows for a large dynamic range of expression levels over which transcripts can be detected and has increased sensitivity for genes expressed at either very high or very low levels when compared to microarrays [36]. The patterns of expression of selected GH-encoding genes between 48 h glucose and 24 h straw was confirmed by qRT-PCR (see Additional file 2) and were in agreement with the RNA-seq data.

**Table 1:** Straw-induced CAZy genes

**Transcript ID**	**Annotation **[18]	**CAZy Family**	**48 h Glucose RPKM**	**24 h Straw RPKM**	**5 h Glucose RPKM**
123818	Endo-β-1,4-xylanase, *xyn2*	GH11	3.26	3776.93	3.72
123989	Cellobiohydrolase, *cbh1*	GH7	1.57	2345.54	2.38
111849	Endo-β-1,4-xylanase, *xyn4*	GH30	1.18	1219.13	0.99
121127	Beta-xylosidase, *bxl1*	GH3	0.85	1182.53	2.15
72526	Alpha-glucuronidase *glr1*	GH67	0.86	1024.64	0.71
72567	Cellobiohydrolase, *cbh2*	GH6	0.58	861.40	0.92
73632	Acetyl xylan esterase, *axe1*	CE5	0.77	676.15	0.56
73643	Copper-dependent monooxygenase, *cel61a*	AA9	0.72	668.44	1.03
123992	Swollenin, contains CBM1, *swo1*	Not determined	3.94	592.13	2.08
74223	Endo-β-1,4-xylanase, *xyn1*	GH11	0.32	500.83	0.05
120749	Beta-glucosidase, *bgl2*	GH1	1.53	476.91	9.38
120961	Copper-dependent monooxygenase, *cel61b*	AA9	0.47	476.22	0.40
120312	Endo-β-1,4-glucanase, *egl2*	GH5	0.36	471.52	0.22
121418	Acetyl Esterase, *aes1*	CE16	0.27	342.73	0.32
69944	Candidate α-xylosidase/α-glucosidase	GH31	0.26	327.82	0.20
73638	Candidate cellulose binding protein, CBM1, *cip1*	Not determined	0.25	323.47	0.41
103049	Candidate endo-polygalacturonase	GH28	0.33	227.26	0.27
76210	Alpha-L-arabinofuranosidase, *abf2*	GH62	0.36	224.30	0.04
54219	Acetyl xylan esterase	CE5	0.04	216.93	0.16
49081	Xyloglucanase, *cel74a*	GH74	1.22	213.92	0.60
122780	Candidate exo-rhamnogalacturonase, *rgx1*	GH28	0.84	130.03	2.04
56996	Beta-mannanase, *man1*	GH5	0.09	108.27	0.15
76672	Beta-glucosidase, *bgl1*	GH3	0.04	105.71	0.22
62166	Beta-mannosidase	GH2	1.88	98.68	4.04
22197	Beta-glucosidase, *cel1b*	GH1	1.11	94.50	1.42
123283	A;lpha-L-arabinofuranosidase I, *abf1*	GH54	0.49	91.97	0.39
122081	Endo-β-1,4-glucanase, *egl1*	GH7	0.03	91.03	0.14
123940	Glucuronoyl esterase, *cip2*	CE15	0.05	87.99	0.13
80240	Beta-galactosidase, *bga1*	GH35	0.61	87.67	0.18
124016	Alpha-galactosidase, *agl2*	GH36	0.76	82.70	0.59
74198	Candidate α-1,2-mannosidase	GH92	0.12	72.91	0.03
112392	Candidate endo-β-1,4-xylanase, *xyn5*	GH11	0.04	64.84	0.10
110894	Candidate endo-β-1,6-galactanase	GH30	0.58	57.01	0.20

Highly expressed CAZy-encoding genes with an RPKM value greater than 50 at 24 h straw and an over 20-fold expression between 48 h glucose and 24 h straw.

Although both fungi seem to use a similar array of GHs, they up-regulate the expression of different CE-encoding genes. Whereas transcripts from CE family 1 were most abundant in *A. niger* (~10% of total CAZy mRNA), transcript levels of genes encoding proteins in CE family 5 were highest in *T. reesei* (~5% of total CAZy mRNA). This is an agreement with previous microarray studies [18]. The genome of *T. reesei* encodes 3 acetyl xylan esterases and 1 cutinase, all belonging to CE family 5 [18]. The highest expression values were recorded for 2 acetyl xylan esterases [JGI:73632, JGI:54219] one of which contains a CBM1 module [JGI:73632]. Acetyl xylan esterases remove acetyl groups at *O*-2 and *O*-3 positions of the xylose chain in arabinoglucuronoxylans [5], a process which has been shown to significantly enhance subsequent hydrolysis of xylans and cellulose [37]. The genome of *A. niger* encodes 3 CE family 1 members: one acetyl xylan esterase, one feruloyl esterase and one un-defined esterase [25]. Expression values of the acetyl xylan esterase and the feruloyl esterase were very high in the presence of straw [3]. Feruloyl esterases cleave ferulic acid groups which are esterified to the 5′-OH of arabinofuranosyl groups (arabinose residues linked to *O*-2 or *O*-3 of xylose) and which can be covalently linked to lignin or other ferulic acid groups in xylans [5]. It appears that the enzyme mix secreted by *A. niger* aids in loosening the lignin-hemicellulose structure in addition to de-acetylating the xylan backbone in order to allow access of other CAZymes to the underlying hemicellulose and cellulose polysaccharides. Thus the bulk of GH and AA enzymes used to degrade straw are from the same GH and AA families in both organisms, whereas different CE family members are used suggesting that both fungi specialised also in the cleavage of different bonds found within plant cell walls.

Not surprisingly, transcript abundance of PL-encoding genes was very low in both fungi (~0.012% of total CAZy mRNA in *T. reesei* and ~0.5% of total CAZy mRNA in *A. niger*), confirming that PLs do not play an important role in wheat straw degradation (as mentioned above). Similar results were obtained through previous microarray studies [18].

*T. reesei* also highly induced the transcription of genes encoding proteins other than hydrolases, which have been proposed to be involved in enhancing cellulose degradation (Table 1). One such enzyme is the expansin-like swollenin, *swo1* (Table 1), thought to play a role in the loosening of the plant cell wall by disrupting hydrogen bonds between plant polysaccharides, thus increasing cell wall area and access of hydrolytic enzymes (such as cellulases) to the underlying polymers [38]. Another enzyme, CIP1 (Table 1), which contains a CBM belonging to family 1, is thought to enhance cellulose hydrolysis [9]. Transcript levels of *cip1* were also detected in the presence of sophorose and regulation of this gene is the same as for other well characterised cellulases (e.g. *cbh1*), indicating a potential role for this protein in cellulose degradation [39]. Our results show that genes encoding both enzymes are highly induced in the presence of straw and that they are regulated similarly to CAZy enzymes-encoding genes, suggesting that these enzymes could be key players in wheat straw degradation. Genes encoding CIP1 and swollenins are absent from the genome of *A. niger*.

Addition of glucose to the straw cultures exerted strong carbon catabolite repression of the CAZy-encoding genes, and CAZy transcript abundance decreased to 0.82% in *T. reesei* (1.4% in *A. niger*, Figure 1) of the total cellular mRNA, with members from GH families 16, 17, 67 and 72 (glucosidases, glucuronidase and glucanosyltransferases) (and GH15 in *A. niger*) being the most expressed CAZy genes under this condition in *T. reesei* (Figure 2).

### Expression of non-CAZy genes in *T. reesei* and comparison with *A. niger*

Thirty-two genes in *T. reesei* which do not encode CAZy enzymes were up-regulated more than 20-fold and reached an RPKM greater than 50 after transfer for 24 hours from glucose to straw-based media (Table 2). In *A. niger*, highly induced genes encoding non-CAZy proteins were divided into 5 broad categories [3]. Interestingly, genes belonging to 4 of these functional categories (lipase, surface interacting proteins, enzymes of the carbon and nitrogen metabolism and transporters) were also highly induced in *T. reesei* suggesting a similar approach for both organisms when responding to the presence of ground wheat straw. Moreover, two more broad functional categories could be defined in *T. reesei* under these criteria: proteins involved in gene regulation and oxidation-related enzymes (Table 2).

**Table 2:** Straw-induced non-CAZy genes

**Transcript ID**	**Annotation**	**48 h Glucose RPKM**	**24 h Straw RPKM**	**5 h Glucose RPKM**
**Lipases**				
64397	Ceramidase family protein, associated to cellulase signal transduction	2.40	95.94	1.08
**Surface interacting proteins**				
119989	HFB2, class II hydrophobin	0.79	503.22	262.84
74282	QI74 orthologue, cell wall protein	0.30	272.50	12.53
123967	HFB3, class II hydrophobin	0.54	182.32	33.50
104277	Cell wall protein, contains HsbA conserved domain	0.15	87.68	0.29
124295	Cell wall protein with a CFEM domain	0.17	58.28	9.80
**Enzymes of carbon and nitrogen metabolism**				
107776	Xylose reductase XYL1	2.79	294.24	4.42
123009	Glutamine synthetase	7.90	214.02	176.34
80340	Alpha1,6-mannosyltransferase	0.18	117.78	3.34
120784	Aldose-1-epimerase	1.43	106.24	1.96
81271	Xylitol dehydrogenase XDH1	3.58	74.44	4.64
49753	L-arabinitol 4-dehydrogenase LAD1	0.07	55.57	0.32
**Transporters**				
3405	MFS (major facilitator superfamily) permease	1.14	1658.36	2.94
50894	MFS permease	0.37	466.75	0.60
104072	Xylose transporter	0.27	324.06	0.30
69957	MFS permease	0.24	314.19	0.48
82309	Predicted MFS transporter	0.47	152.99	0.17
120017	Oligopeptide transporter	1.84	141.28	8.13
106330	MFS transporter	0.52	92.17	0.65
50618	MFS transporter	0.14	88.17	0.58
38812	Iron transporter	0.66	68.02	5.06
80863	MFS transporter	1.80	67.87	9.25
46794	Oligopeptide transporter	0.17	56.32	3.48
**Gene regulation**				
44747	SNF2 family helicase	6.21	228.14	23.38
80980	Peptidyl arginine deiminase	0.09	118.95	10.07
108357	C_2_H_2_ transcriptional regulator	0.62	108.73	0.65
**Oxidation-related**				
80659	Alcohol oxidase AOX1	0.95	1618.88	1.03
56840	GFO_IDH_MocA dehydrogenase	1.93	407.87	1.56
76696	Flavin-containing monooxygenase	0.67	111.38	0.49
123827	Bifunctional catalase/peroxidase	4.13	105.08	6.58
22771	Dehydrogenase	2.01	79.27	1.02
123978	Methanol oxidase	0.21	61.55	0.24

Highly expressed non-CAZy-encoding genes with an RPKM value greater than 50 at 24 h straw and an over 20-fold expression between 48 h glucose and 24 h straw.

#### Lipases

In *T. reesei*, one lipase-encoding gene had a similar transcriptional pattern to many GH and CE-encoding genes. The gene contains a secretion signal and encodes a protein belonging to the neutral/alkaline non-lysosomal ceramidase family which hydrolyse the sphingolipid ceramide into sphingosine and free fatty acid, bioactive lipids serving as cellular messengers [40]. The alkaline ceramidase described here is thought to be involved in cellulase signal transduction [41]. NCBI BLASTP analysis revealed the protein encoded by this gene [JGI:64397] to be highly similar to a ceramidase [JGI:120161] in *A. niger*, which was also highly induced in the presence of straw [3]. It is possible that this ceramidase is secreted with, and maybe regulated in a similar manner to the CAZy enzymes and participates in wheat straw deconstruction, although expression of this gene in the presence of other lignocellulosic substrates requires further characterisation. This gene presents an interesting candidate for future studies on enzymes which could be involved in plant cell wall degradation.

#### Hydrophobic surface interacting proteins

Five genes encoding two hydrophobins and three cell wall proteins were induced in *T. reesei* when switching from glucose to straw and all genes were repressed when glucose was added to the cultures. These genes have a transcriptional profile similar to many genes of the CAZy group. Hydrophobins can adsorb to hydrophobic surfaces and to interfaces between hydrophobic and hydrophilic phases, thus mediating the interaction of the fungus with its environment [42,43]. In *Aspergillus oryzae*, the hydrophobin RolA recruits the esterase CutL to the synthetic polyester polybutylene succinate-*co*-adipate (PBSA) and promotes its degradation [43]. A similar role for the two highly expressed hydrophobins of *T. reesei* could therefore be envisaged, as has been proposed for two *A. niger* hydrophobin-encoding genes which were also highly induced in the same conditions [3]. The other three highly induced cell wall protein-encoding genes are likely to also have a role in mediating interactions of the fungus with a solid substrate. The QI74 cell wall protein-encoding gene has been shown to be induced in *Trichoderma harzianium* when replacing glucose medium with chitin, simulating mycoparasitic conditions [44]. NCBI BLASTP analysis of the protein encoded by the gene with transcript ID 104277 (Table 2) revealed the presence of putative conserved domain which is also found in the HsbA (hydrophobic surface binding protein A) superfamily; an enzyme family which differs in structure and mechanism of surface interaction, when compared to hydrophobins [43]. In *A. oryzae*, HsbA also recruits the cutinase CutL to PBSA to promote its degradation [43]. The third gene [JGI:124295] described here, encodes a cell wall protein with a predicted extracellular CFEM (conserved fungal specific extracellular membrane-spanning) domain. Some Pth11-like GPCRs (G-protein coupled receptors) have associated CFEM domains and are important for sensing surface cues [45,46]. Pth11-like GPCRs were first described in *Magnaporthe grisea*, where they play an essential role in plant host invasion and pathogenicity [45,46]. The observation of the induction of hydrophobic surface interacting protein-encoding genes in both *T. reesei* and *A. niger* suggests that the recognition of solid surfaces is an important step in the fungal response to the plant cell wall and further experiments are required to confirm this. Nevertheless, genes encoding hydrophobic surface interacting proteins present interesting candidates which potential roles in enhancing lignocellulose degradation.

#### Transporters

Eleven genes, encoding seven transporters of the major facilitator superfamily (MFS), one xylose transporter, two oligo-peptide transporters and one iron transporter were highly transcribed in straw and repressed in glucose-rich conditions. The MFS superfamily is a large family of transporters which can be divided into a further 17 families of which families 1, 5 and 7 mediate monosaccharide (hexoses, pentoses) and oligosaccharide transport into the cell by coupling it to proton symport or antiport [47]. The first gene listed in Table 2 [JGI:3405] is possibly involved in hexose and disaccharide transport as NCBI BLASTP analysis of the encoded protein [JGI:3405] revealed 75% amino acid sequence identity to a hexose transporter from *Glomerella graminicola*[48] and 74% identity to a lactose permease from *Verticillium dahlia*. This transporter may belong to family 1 of MFS transporters which couple sugar uptake to proton symport and which are involved in the uptake of galactose, quinate, lactose, maltose and α-glycosides [47]. NCBI BLASTP analysis of the protein [JGI:50894] encoded by the second gene listed in Table 2 [JGI:50894] revealed 76% sequence identity to a high affinity glucose transporter from *Gaeumannomyces graminis* and it may belong to family 7 of MFS transporters which couple hexose to proton import and are involved in the uptake of fucose, galactose and glucose [47]. The induction of transporter-encoding genes after 24 h in the presence of straw indicates that the cellulose and hemicellulose fractions of the wheat straw are being degraded, subsequently releasing simple sugars which are taken up by the fungus.

#### Carbon metabolism

As in *A. niger*[3], genes of the xylose utilisation pathway such as xylose reductase XYL1 (Table 2) and xylitol dehydrogenase XDH1 (Table 2) were up-regulated more than 20-fold (Table 2) when switching from glucose to straw. XYL1 also reduces the pentose sugar L-arabinose to L-arabinitol which is then further oxidised to L-xylulose by the L-arabinitol dehydrogenase LAD1 (Table 2) [49]. In addition, LAD1 is also involved in the alternative pathway of D-galactose metabolism whereas aldose-1-epimerase (Table 2) catalyses the first step of the Leloir pathway of D-galactose catabolism [50]. This indicates that after 24 h, *T. reesei* had internalised hemicellulosic sugars such as xylose, arabinose and galactose which had then entered their respective metabolic pathways.

### Antisense transcription

Regulation of gene expression can also occur at the post-transcriptional level through regulatory RNAs. Natural antisense transcripts (NATs) are non-protein coding, fully processed mRNAs, which can partially overlap the protein-coding transcripts and which have many regulatory roles [51]. NATs have been found in several fungi including *A. niger* and *N. crassa*[3,52]. To calculate the levels within our *T. reesei* transcriptomes, the number of reads corresponding to the non-coding strand was counted for each gene and AS (antisense) RPKM values were calculated in each condition. Approximately 1.82%, 1.47% and 2.79% of all reads in *T. reesei* were antisense reads when mycelia were grown in 48 h glucose, 24 h straw and 24 h straw + 5 h glucose respectively. Thus, AS transcription was detected in *T. reesei* and 630 genes had an AS RPKM > 1 in at least one condition (see Additional file 3). The 630 genes encoded proteins involved in a wide variety of cellular functions. The% of AS transcripts was similar in *T. reesei* when compared to *A. niger*[3].

The ratio of antisense:sense expression under glucose 48 h and straw 24 h conditions was calculated for these 630 genes in order to find genes with AS transcripts which change between the two conditions. Most of these genes have a low AS/S ratio on 24 h straw indicating that sense transcripts dominate over AS reads in this condition.

**Figure 3:**
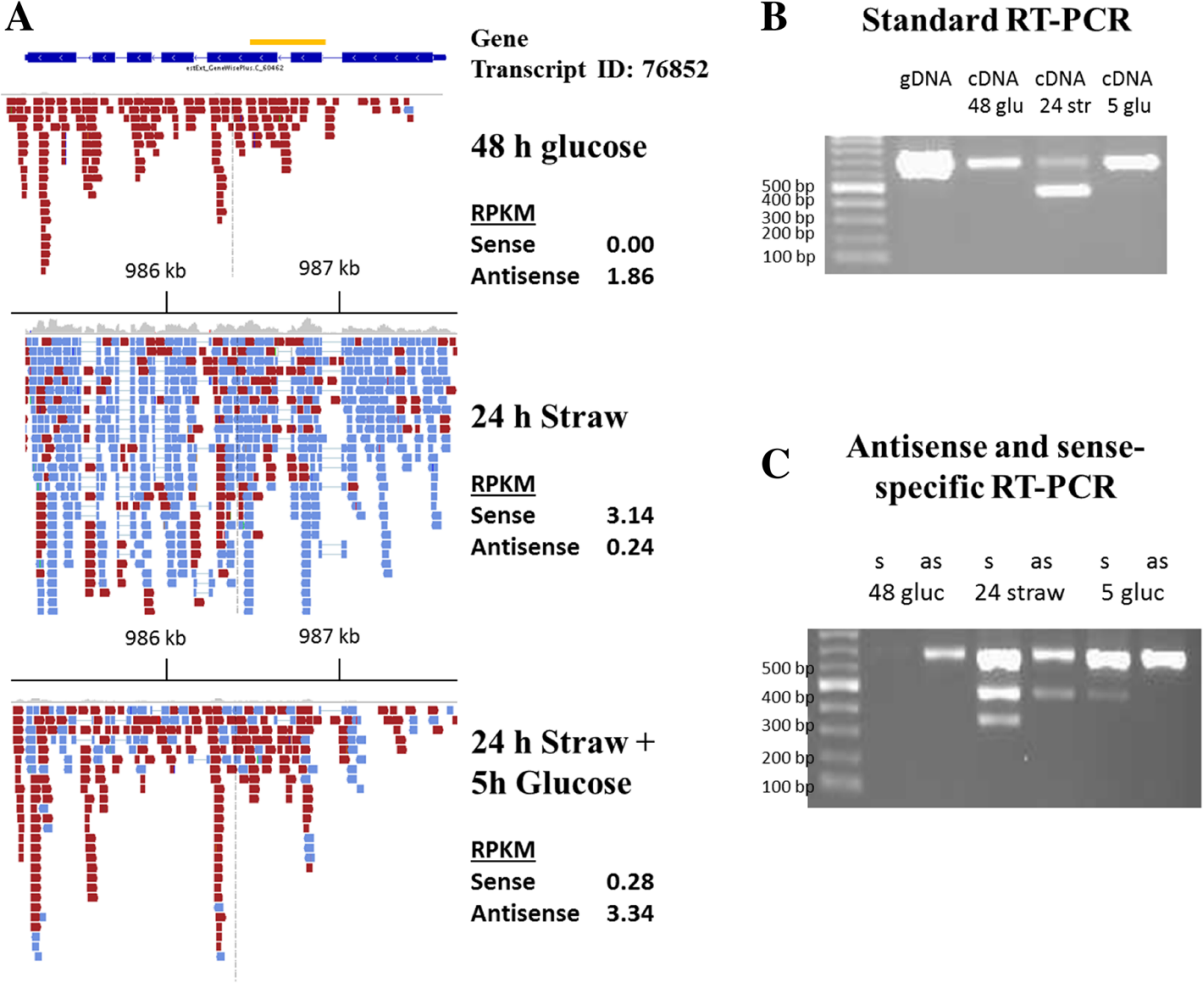
**Sense and antisense transcription of gene with ID 76852. (A)** IGV (Integrative Genome Viewer) output of the alignment of RNA-seq reads to the genome region corresponding to gene transcript ID 76852 under each condition. Blue reads represent sense RNAs, red reads represent antisense RNAs. **(B)** RT-PCR using gene specific primers (indicated by a yellow line) on oligo(dT) primed cDNA. The expected band size from spliced, sense transcripts is 476 bp and the size of the non-spliced antisense transcripts is 670 bp and has the same size as products from reactions run on gDNA. **(C)** Strand-specific RT-PCR using one of the standard PCR primers to synthesise cDNA from one strand only and then the PCR step was performed by using the same primer together with the opposing gene-specific primer. In 24 h straw and 5 h glucose both antisense (extend differently over both introns) and sense (differently spliced introns) transcripts are present, explaining the presence of multiple PCR products.

Confirmation of the presence of NATs in *T. reesei* was achieved by strand-specific PCR analysis for a gene [JGI:76852] with NATs (Figure 3). This gene is predicted to encode a secreted β-glucuronidase, belonging to GH family 2. Strand-specific RT-PCR confirmed the presence of spliced and non-spliced S and AS transcripts of different sizes (Figure 3B and C) in all three conditions as was previously reported for a gene containing NATs in *A. niger*[3]. Regulation at the post-transcriptional level presents an interesting area for further research.

## Conclusions

This study explored the mechanisms used by *T. reesei* to degrade an industrially-relevant substrate (wheat straw) for the generation of biofuels and made comparison with the strategies employed by *A. niger*[3]. Both fungi encode a mix of predicted GHs, CEs and AAs and some families are species-specific. In the presence of wheat straw, many of the CAZy protein-encoding genes were up-regulated in the two fungi and repressed in both glucose conditions. *T. reesei* and *A. niger* use a set of core enzymes from the same GH (3, 7, 11, 30 and 67) and AA (9) families but from different CE families for wheat straw degradation. The amount of CAZy mRNA (as a proportion of total cellular RNA) in *T. reesei* after 24 h incubation in straw was less than that in *A. niger* (number of CAZy-encoding genes represent ~ 2.5% of the coding genome in both organisms). Thirty-two genes encoding non-CAZy enzymes in *T. reesei* had an expression pattern similar to the CAZy-encoding genes. The majority of these genes could be classed into functional categories which were also described in *A. niger*[3], suggesting a similar approach for both fungi to the degradation of a solid, lignocellulosic substrate. Furthermore, as described in *A. niger*[3], the existence of NATs (a type of regulatory RNA) were also shown to be present in *T. reesei*. This presents an interesting area for future research as regulatory RNAs may be involved in the post-transcriptional regulation of genes encoding enzymes involved in carbohydrate degradation. The use of Next Generation RNA sequencing has allowed us to gain preliminary insights into the global gene expression profile in response to a complex lignocellulosic substrate and to identify genes in *T. reesei*, which encode enzymes which have previously not been associated to lignocellulose deconstruction. Furthermore we have confirmed the presence of a type of regulatory RNAs which have not previously been described in *T. reesei* yet.

## Methods

### Strains and growth conditions

*T. reesei* QM6a [53,54] was used throughout this project. Conidia were produced from glycerol stocks of *T. reesei* grown on potato dextrose agar medium (PDA: 4.0 g/L potato extract, 15.0 g/L agar, 20.0 g/L) at 28°C. Conidia were harvested with 2 ml 0.01% (w/v) Tween 80. Liquid batch cultures were inoculated at a concentration of 10^5^ spores/mL.

Strains were cultured in 100 ml *Trichoderma* Minimal Media [TMM: 15 g/L KH_2_PO_4_, 5 g/L (NH_4_)_2_SO_4_, 10 g/L of the respective carbon source, 0.005 g/L FeSO_4_.7H_2_0, 0.0016 g/L MnSO_4_.H_2_0, 0.0014 g/L Zn.SO_4_.H_2_0, 0.0037 g/L CoCl_12_.6H_2_0, 0.6 g/L MgSO_4_, 0.6 g CaCl_2_] in 250 mL Erlenmeyer flasks at 28°C, shaking at 150 rpm. Mycelia were grown for 48 h in 1% (w/v) glucose, after which they were removed by filtration through Miracloth (Merck), washed with double-distilled water (ddH_2_O), and transferred to fresh media supplemented with the relevant carbon source at 1% w/v. Three different sets of conditions were distinguished: 1) growth from conidia for 48 h in the presence of glucose as the sole carbon source (48 h glucose), 2) 24 h after transfer of washed mycelia from 1) into media containing ground wheat straw as the sole carbon source (24 h straw) and 3) 5 h after addition of glucose to the straw cultures from 2) (5 h glucose). Transcriptomes were analysed from triplicate cultures of 48 h glucose and 24 h straw and from duplicate cultures of 5 h glucose.

Ball milling, sugar, lignin and crystallinity analysis of the wheat straw used in this study can be found elsewhere [3].

### RNA extraction

Mycelia from each condition were snap-frozen and ground to a fine powder under liquid N_2_ using a mortar and pestle. 100 mg of mycelial powder was used for RNA extraction, the procedure of which was described elsewhere [3]. Briefly, total RNA was extracted from mycelial powder using TriZol reagent (Invitrogen) according to manufacturer’s instructions. Extracted RNA was purified using the Qiagen RNeasy Mini Kit following the manufacturer’s instructions of the RNA clean-up protocol with on-column DNA digestion.

### RT-PCR and qRT-PCR

The synthesis of cDNA was carried out as described by [3]. PCR reactions were performed using RedTaq DNA Polymerase (Sigma) and 1 μL of cDNA in a 20 μL reaction. PCR conditions were 30 cycles of denaturation at 94°C for 1 min, annealing at 60°C for 2 min and extension at 72°C for 3 min. Strand-specific PCRs (ssPCRs; as described in [3]) were carried out at an annealing temperature of 64°C using the primer pairs indicated in the Additional file 4.

qRT-PCR reactions were run using the same system as described by [3] and carried out for 40 cycles with denaturation at 95°C for 30 s and annealing at 64°C for 30 s and extension at 60°C for 60 s. Briefly, qRT-PCR reactions were run in triplicates per gene in each condition in a total reaction volume of 20 μL containing 11.0 μl Fast SYBR green master mix (Applied Biosystems), 0.11 μL/primer (175 nM final concentration), 2.2 μL of cDNA and 8.58 μL ddH_2_O. Gene expression values were calculated against a standard curve of known genomic DNA concentrations. All primer pair sequences are listed in the Additional file 4.

### RNA-seq and subsequent data analysis

Ribosomal RNA was degraded in 10 μg of total RNA using the Ribominus Eukaryotic kit (Invitrogen). SOLiD whole transcriptome libraries were made according to the SOLiD Whole transcriptome kit protocol (Applied Biosystems) and the library concentrations were measured with the Quant-it HS dsDNA assay kit (Invitrogen). Libraries were pooled to equimolar amounts (Invitrogen) and gel purified using 2% size-select E-gels to 200–300 bp (Invitrogen). Emulsion PCR (0.5 M final concentration of pooled libraries) and bead-based enrichment was done according to the SOLiD 4 Templated bead preparation guide containing library. Sequencing was carried out on a SOLiD 4 ABi sequencer platform according to manufacturer’s instructions to generate single fragment 50 bp colour space reads.

Using the BioScope version 1.3 Whole Transcriptome Pipeline (LifeTechnologies), reads from each SOLiD 4 libraries were initially filtered against sequencing library adaptors and other sequencing artefacts. Reads were then mapped independently to the entire unmasked and masked versions of the annotated genome assembly of *T. reesei*, and to defined gene transcript sequences (JGI *T. reesei* assembly version 2, annotation filtered models version 2.0). For mapping against the genome assemblies, it was also possible to align reads against a library of exon junctions derived from the exon coordinates detailed in the genome annotation. This allowed reads spanning exon boundaries to be included in alignment results and be recorded in BAM format. Subsequently, the reads corresponding to rRNA encoding gene regions were removed from the BAM records before further downstream analysis. The resulting BAM file of mapped reads from each sample against the unmasked and masked genomes were processed with HTseq-count [3] to generate read counts per gene from uniquely aligned reads. These counts were determined for both the sense strand only and to both strands (unstranded). Summary metrics for these results are shown in Additional file 5.

When comparing the read alignment metrics between the unmasked and masked genome assemblies a negligible difference of less than 1% is seen for total mapped reads, uniquely mapped reads and for reads mapped within annotated genes. This suggests that the masked regions of the genome do not correspond to transcribed genomic regions. The percentage of reads that were uniquely mapped to the unmasked assembly, as a proportion of total mapped reads, was 63.60% for all 48 h glucose replicates, 69.64% for all 24 h straw replicates and 52.95% for the 5 h glucose duplicates (Additional file 5).

For transcript sequence read mapping, less than half the total number of mapped reads compared to the mapping against the annotated genome was observed for all conditions. This can be explained by the difference in reference sequence space used in mapping. Transcript sequences were derived from the annotated exon coordinates within the genome sequence; therefore it would not be possible to map reads corresponding to transcribed regions outside of these defined coordinates. These unmapped reads could be attributed to incomplete annotation of the genome and/or to reads that are not mRNA coding. This is supported by a similar number of uniquely mapped reads to the transcripts within gene coordinates of the genome mapping when compared to the transcripts. The percentage of reads mapped uniquely to transcripts sequences, as a proportion of total transcript mapped reads, was 38.49%, 54.93% and 33.83% for the conditions 48 h glucose, 24 h straw and 5 h glucose respectively (Additional file 5).

For gene expression analysis the mapped read counts per gene calculated against the unmasked genome. Counts were determined for both sense strand only and to both strands (unstranded), as described previously. Antisense read counts per gene were calculated by subtracting the sense counts from the other. Read counts were then normalized to RPKM (Reads Per Kilobase per Million mapped reads) expression values for each gene [3] and visualised with the Integrative Genome Viewer (IGV 1.5) programme [3].

Differential expression values were determined using DEGseq [23] using sense read counts per gene for each experimental condition. DEGseq implements three independent statistical tests (Fisher’s Exact Test [21], Likelihood Ratio Test [22] and an MA-plot-based method with the Random Sampling method [23]).

### Availability of supporting data

The raw and processed RNA-sequencing data sets supporting the results of this article are available in the NCBI’s Gene Expression Omnibus [55] repository under GEO accession number GSE44648 [http://www.ncbi.nlm.nih.gov/geo/query/acc.cgi?acc=GSE44648].

## Abbreviations

AA: Auxiliary Activities; AS: AntiSense; CAZy: Carbohydrate Active enzyme; CBM: Cellulose Binding Module; CE: Carbohydrate Esterase; CFEM: Conserved fungal specific extracellular membrane-spanning; GH: Glycoside Hydrolase; GPCR: G-Protein Coupled Receptor; MFS: Major Facilitator Superfamily; NAT: Natural Antisense Transcript; PL: Polysaccharide Lyase; RPKM: Reads Per Kilobase of exon model per Million mapped reads; S: Sense.

## Competing interests

The authors declare that they have no competing interests.

## Authors’ contributions

SD, STP and DBA conceived and designed the experiments. LR and SM carried out the experiments. LR, SD, STP, MJB and DBA analysed the data. MJB contributed the reagents/materials/analysis tools. LR, SD, STP and DBA wrote the paper. All authors read and approved the final manuscript.

## Supplementary Material

Additional file 1 **Individual gene sequencing information.** Mapped reads and RPKM values for the three biological replicates and the combined values of all three replicates as well as statistical significance scores for all genes at 48 h glucose, 24 h straw and 24 h straw + 5 h glucose. Click here for file

Additional file 2 **qRT-PCR of GH-encoding genes.** Transcript levels of genes encoding one GH family 7 protein (CBH1, [JGI:123989]), one GH family 11 protein (XYN2, [JGI:123818]) and two GH family 61 proteins (CEL61A [JGI:73643] and CEL61B [JGI:12961]) in mycelia grown for 48 h in glucose-based medium and then transferred into media containing straw as the sole carbon source for 24 h. Error bars indicate the standard deviation for three replicates and * indicates significant difference (a p-value of <0.0001 in an equal variance, one-tailed *T* test) between transcript levels at 48 h glucose and 24 h straw. Click here for file

Additional file 3 **Antisense RNA.** Antisense and sense RPKM values and ratios for all genes with an antisense RPKM >1 in either 48 h glucose, 24 h straw and/or 24 h straw + 5 h glucose. Click here for file

Additional file 4 **Primer pair details.** Sequences, annealing temperatures and predicted gene product sizes. Click here for file

Additional file 5 **Summary metrics of the number of reads.** Total number of reads, total number of filtered reads, amount of mapped and uniquely mapped reads to both DNA strands (stranded and unstranded), percentage of total mapped reads and percentage of uniquely mapped reads of total mapped reads corresponding to the *T. reesei* unmasked genome, masked genome and transcript sequences for each sample. Click here for file
