# Supplementary figures and images for: Genome-wide transcriptional response of Trichoderma reesei to lignocellulose using RNA sequencing and comparison with Aspergillus niger
Source: BMC Genomics. 2013 Aug 9;14:541. doi: 10.1186/1471-2164-14-541 (PMC3750697; doi:10.1186/1471-2164-14-541)

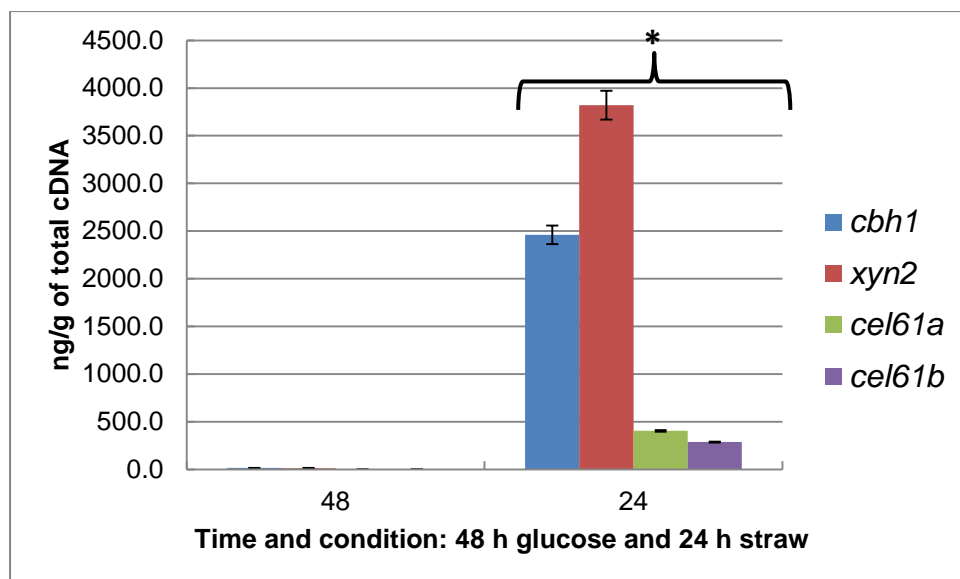

Supplement: Additional file 2 — qRT-PCR of GH-encoding genes. Transcript levels of genes encoding one GH family 7 protein (CBH1, [JGI:123989]), one GH family 11 protein (XYN2, [JGI:123818]) and two GH family 61 proteins (CEL61A [JGI:73643] and CEL61B [JGI:12961]) in mycelia grown for 48 h in glucose-based medium and then transferred into media containing straw as the sole carbon source for 24 h. Error bars indicate the standard deviation for three replicates and * indicates significant difference (a p-value of <0.0001 in an equal variance, one-tailed T test) between transcript levels at 48 h glucose and 24 h straw. [file 1471-2164-14-541-S2.pdf]
