# Supplementary material for: Genome-wide transcriptional response of Trichoderma reesei to lignocellulose using RNA sequencing and comparison with Aspergillus niger
Source: BMC Genomics. 2013 Aug 9;14:541. doi: 10.1186/1471-2164-14-541 (PMC3750697; doi:10.1186/1471-2164-14-541)
Supplement: Additional file 4 — Primer pair details. Sequences, annealing temperatures and predicted gene product sizes. [file 1471-2164-14-541-S4.pdf]

Name	Forward oligo (5’ to 3’)	Annealing temperature (°C)	Reverse oligo (5’ to 3’)	Annealing temperature (°C)	Predicted Product size (bp)	Predicted Product size (bp) no intron
cbh1	cagacaagggcggcctgactc	60.6	cgctgtagccaataccgccg	60.1	459	396
xyn2	cgggcagttctccgtcaactg	59.2	ggacgggttgtaggtgccaaag	59.3	202	141
gh61a	agtagtgggctggacggctgc	60.1	ccgagcctgagacggcaatg	60.2	521	467
gh61b	cctgcgctgggtcagattcag	60.5	cgagcctgtgacggcgatgt	60.6	294	226
